# Acceptability of breast cancer risk assessment for women aged 30–49: Views of women from six under-served groups

**DOI:** 10.1371/journal.pone.0356263

**Published:** 2026-08-17

**Authors:** Victoria G. Woof, Maria Valasaki, Francisca Stutzin Donoso, Lorna McWilliams, Helen Morley, Juliet Usher-Smith, David P. French

**Affiliations:** 1 Manchester Centre for Health Psychology, Division of Psychology and Mental Health, University of Manchester, Manchester, United Kingdom; 2 The Primary Care Unit, Department of Public Health and Primary Care, University of Cambridge, Forvie Site, Cambridge, United Kingdom; Touro University California College of Pharmacy, UNITED STATES OF AMERICA

## Abstract

Inequalities in breast screening uptake and cancer outcomes persist among under-served women. To reduce disparities, it is essential to understand women’s views when developing proactive early detection and prevention services. This study explored the acceptability of introducing a breast cancer risk assessment service for women aged 30–49, offering early screening and/or risk-reducing medication to those at moderate or high-risk. We conducted extensive engagement with six communities of typically under-served women, selected to represent diverse and overlapping sources of disadvantage: (1) South Asian Muslim women, (2) Black women, (3) Roma women, (4) women in low socio-economic neighbourhoods, (5) women with learning disabilities and/or autism, and (6) women with long-term anxiety and/or depression. Fifty-two women participated in 10 focus groups and one interview. Data were analysed using a thematic framework approach. All groups were enthusiastic about a breast cancer risk assessment for young women. Across groups, female healthcare staff, local delivery, a straightforward process, and assurances about data privacy were valued to reduce barriers and promote engagement. Women also supported local events to promote risk assessment and breast cancer awareness. Concerns about the emotional impact of risk information highlighted the need for clear communication and tailored support across groups. Among ethnic minority women, breast cancer was often seen as taboo, limiting discussion and complicating the sharing of family history. These findings highlight that under-served women face both shared and unique challenges. To enhance uptake and reduce longstanding early detection inequalities, risk assessment services should be culturally sensitive, streamlined, and supported by community-based awareness initiatives.

## Introduction

Globally, the incidence of breast cancer in pre-menopausal women is increasing [1,2]. In the UK, approximately 7% of all breast cancer cases occur in women under age 40 years [3]. Compared to cases diagnosed in women over age 50, breast cancers in younger women tend to be more aggressive, with a ten-year survival rate of 70% for those aged 40 and below, compared to 87% for women aged 50 and above [4]. Given this, there is increasing interest in approaches to identify pre-menopausal women at increased risk of developing the disease to support prevention and improve survival through early detection and treatment [5].

In the UK, the NHS Breast Screening Programme (NHSBSP) offers screening every three years to women aged 50–71. However, in line with UK National Institute for Health and Care Excellence (NICE) guidelines, younger women who self-present to primary care with a strong family history of breast cancer are offered a referral to family history risk and prevention clinics to assess their eligibility for early breast screening and/or preventative medication [6]. Nevertheless, around 65% of breast cancers diagnosed in younger women occur in those without a family history [4,7]. Given this, to better identify younger women at increased risk, the feasibility of personalised breast cancer risk assessment is being investigated [5]. Risk prediction models such as the Tyrer-Cuzick [8] and BOADICEA [9,10] can estimate an individual’s likelihood of developing breast cancer by incorporating factors such as family history, hormonal and reproductive factors, polygenic risk scores, and health behaviours. This area has progressed as far as initial feasibility trials offering risk assessments to women aged 30–49, laying the groundwork for broader implementation [11–13]. However, addressing potential inequalities of uptake has been identified as a key issue that needs to be resolved before any service is implemented [5,14].

In the NHSBSP, uptake of mammography is consistently lower among women facing social, cultural, or health-related disadvantages [15–17]. For example, women living in areas of social deprivation may encounter barriers such as low health literacy, financial constraints, competing life pressures, and logistical challenges associated with the location of screening sites [16,18,19]. Similar challenges are also observed among women with learning disabilities, where low health literacy and inaccessible invitation methods further hinder participation [20,21]. Women from minoritised communities, such as South Asian women, and those from Gypsy, Irish Traveller, and Roma communities, may additionally experience disadvantages related to stigma, fear, limited knowledge of screening programmes, and language barriers that restrict access [22–24]. Women with mental health conditions also experience reduced screening uptake, often linked to difficulties accessing primary care, diagnostic overshadowing, and socioeconomic disadvantage [25]. Across these groups, overlapping and intersecting barriers contribute to persistent inequalities in breast screening participation, which could contribute to delayed diagnoses, poorer health outcomes, and preventable mortality.

Qualitative research to date suggests that young women generally find the implementation of breast cancer risk assessment acceptable, provided the service is easily accessible, well-resourced, and supported by appropriate healthcare expertise [26]. However, while some perspectives from women from under-served backgrounds have been included, most participants in this study were White-British and from middle to high income areas. Similarly, trials testing the feasibility of introducing risk assessment at population screening level (aged 50–70) have struggled to engage women from underserved groups, with participants disproportionately identifying as White-British and from medium to high income areas [27]. Therefore, these gaps highlight an urgent need to engage more meaningfully with women from under-served communities before implementing any new health initiative, to ensure services are equitable and responsive to diverse needs.

This study aimed to explore the perceived acceptability of breast cancer risk assessment among women aged 30–49, with a particular focus on those from under-served communities and those experiencing barriers to accessing healthcare services, contributing to health inequalities. This study also aimed to explore where these women’s experiences of healthcare inequality overlap and where they diverge to inform the development of a service which addresses significant areas of marginalisation. This research forms part of a funded multi-study project designed to develop and evaluate an inclusive risk assessment service for young women.

## Methods

### Design

A cross-sectional qualitative design was used, employing focus groups and one-to-one semi-structured interviews. Data collection took place either in-person and online via Zoom.

### Participants and recruitment

Women aged between 30–49 without a history of breast cancer from the following communities and backgrounds were approached to participate:

- women from South Asian (Muslim) backgrounds,
- women from Black African and Black Caribbean backgrounds,
- women from the Roma community,
- women from low socio-economic neighbourhoods (based on postcode data),
- women with self-reported learning disabilities and/or autism and,
- women with self-reported long-term anxiety and/or depression (long-term defined as a diagnosis of 1-year or longer).

These groups were identified based on available evidence and stakeholder consultations suggesting these communities of women face significant barriers to breast screening and are underrepresented in breast cancer risk assessment research. They were selected to capture a range of disadvantage, ensuring that the major reasons for lack of uptake could be included. The choice to include different groups of women was endorsed through Patient and Public Involvement and Engagement (PPIE) conducted prior to submission of the grant, although participants did not suggest the selected groups themselves. In addition, the groups partly reflect communities with whom the researchers have previously worked with given our interest as researchers on reducing health inequalities. The researchers also acknowledge the presence of intersectionality within these groups, recognising that women may belong to multiple groups depending on their identities and experiences.

Recruitment strategies for each group were developed in collaboration with the study’s PPIE contributors who represented their communities, as well as professionals working in roles that support them. These contributors advised on the relevance of the research question and topic area and guided the research team on effective ways to approach women to participate.

Acting on this guidance, the research team approached a range of charities/community organisations in Greater Manchester and the Cambridgeshire area. VGW and MV initiated contact both in-person and via email to assess interest in supporting recruitment. The researchers then met or had an email exchange with charity/community organisation representatives to discuss the study’s relevance to their communities and design tailored communication strategies for recruitment.

In some cases, posters in community spaces and information shared via mailing lists proved sufficient. In others, particularly for women whose first language was not English, or for women with learning disabilities and/or autism, trusted community leaders and support workers discussed the research directly with potential participants. This allowed women to hear about the study from someone familiar in a trusted setting. To minimise any risk of coercion, women were encouraged to contact the lead researchers directly, or, if they preferred, to have a trusted contact from the charity/community organisation reach out on their behalf.

Recruitment proved challenging for the long-term anxiety and/or depression group, with only 4 of the 18 charities contacted sharing the study information within their networks. Consequently, low participant numbers led researchers to draw on professional networks to recruit additional participants.

Following the advertisement of the study, women who were interested in participating and who met the eligibility criteria registered their interest either through staff at the charity/community organisation or directly with the lead researchers (VGW & MV) via telephone or email. Eligible women who met two or more criteria and were eligible to participate in one or more of the focus groups were asked to indicate their preferred focus group for participation. For example, women from low socio-economic neighbours who also identified as South Asian (Muslim) were asked which focus group they would prefer to participate in. Women who preferred or who were unable to participate in a focus group were offered a one-to-one interview either in-person or online via Zoom. Recruitment for the study ran from March to April 2025.

### Procedure

Focus groups were selected as the preferred method of data collection because they provide an environment that fosters dynamic discussion and exploration of differing viewpoints, allowing for a comprehensive examination of the topic [28]. Focus groups took place either online via Zoom or in-person in community spaces known and local to women.

At each focus group, two female researchers were present so that one acted as the moderator, while the other took notes of the discussion to feedback to the group. Before the discussion began, participants provided written or verbal informed consent, including for data collection to be audio-recorded, and completed optional demographic questions (S1 File). Capacity for consent for women with learning disabilities and/or autism was determined in collaboration with support workers and charity staff, and the researcher took time to explain the consent process thoroughly and discuss any questions or concerns women had. They were then reminded of the discussion topics, given an overview of the group’s ground rules, and provided with time to ask questions. To facilitate a comfortable atmosphere, icebreaker activities were conducted, allowing both participants and researchers to get to know each other. Once this was completed, the audio recorder was turned on, and the focus group began.

A semi-structured topic guide was used flexibly throughout (S1 File). The topic guide was initially developed by the research team and informed by the Theoretical Framework of Acceptability (TFA); a tool used to evaluate the acceptability of healthcare interventions [29]. The topic guide was then further refined in collaboration with the study’s PPIE volunteers. The topic guide underwent several revisions before the final version was agreed upon. Topics included, how women discuss breast cancer and risk within their communities, their thoughts on the offer of a breast cancer risk assessment, potential barriers to accessing the assessment, as well as their views on the assessment process, including the provision of information and communication. Questions also explored views on breast cancer early detection and prevention management.

The primary topic guide was adapted slightly for those with learning disabilities and/or autism and the Roma community group, following feedback from PPIE contributors, support workers and charity/community organisation staff (S1 File). These modifications were made to enhance clarity and ensure cultural appropriateness. Breaks were provided in all focus groups when required. During this time the audio recorder was paused.

For South Asian (Muslim) and Roma women, many of whom did not speak English as their first language, focus groups were conducted in Bengali and Romanian. Interpreters received the topic guide in advance to familiarise themselves with the subject matter and the questions to be discussed. VGW met with the interpreters prior to the focus groups to review the subject matter and clarify any terminology that required further explanation into either language. During these focus groups, the researcher addressed the women directly in English, while the interpreter translated and provided English translation to the researcher in the third person. With advice from the Roma community contact, a considerable amount of time was also spent raising awareness about the signs and symptoms of breast cancer, as well as risk factors during relevant points in the focus groups. This was done on the advice of the Roma community contact who disclosed that many of the women taking part had little to know knowledge about breast cancer and some time building awareness would help to contextualise risk assessment for the women in order for them to answer the topic guide questions.

For the two focus groups held with women with learning disabilities and/or autism, participants were encouraged to bring a carer or support worker to assist them. To accommodate the needs of these women, more frequent breaks were incorporated, and in some instances, carers or support workers rephrased the questions to support the women in providing their answers. For some questions traffic light cards were used to gauge opinion and as prompts to explore reasons behind the colours chosen. As with the Roma community, time was also spent to raise awareness about breast cancer symptoms and risk factors to help contextualise risk assessment for those with limited knowledge about breast cancer.

For one participant who was unable to attend the focus group with women with anxiety and/or depression, a one-to-one semi-structured interview was conducted by MV via Zoom. The same consenting procedures and primary topic guide were used.

At the end of each focus group and interview, women were provided with a debrief sheet which detailed more information about the study, as well as useful resources should women have had any concerns about breast cancer. Women were also provided information leaflets on the signs and symptoms of breast cancer. These leaflets were available in multiple languages and easy read format. All women were also compensated for their time.

All data were transcribed verbatim by an external transcription agency, which were checked by VGW for accuracy against the original audio recording and amended where needed. Identifiable information was anonymised and each participant allocated a pseudonym.

### Ethical approval

This research was approved by the University of Manchester Research Ethics Committee 1 (UREC1; Ref: 2024-21249-38554). All participants provide written consent.

### Analysis

Following data collection, the research team decided that data from each of the six groups of women would be analysed together. While these communities are diverse, they do share common experiences of marginalisation and inequalities in healthcare access. Analysing experiences collectively meant the research team were able to examine to what extent women shared views on introducing a breast cancer risk assessment for young women, as well as the unique differences and challenges women face within each group.

Data were analysed in NVivo12 using thematic analysis, managed using the framework approach [30]. Primary data analysis was conducted by VGW and refined by MV, FSD and DPF. Analysis began with familiarisation, with VGW listening to audio recordings and reading transcripts of the focus groups and interview. Coding was both inductive and deductive, with the initial framework considering the domains of the TFA [29] but not being used deductively. Four transcripts were initially coded by VGW and an initial coding framework was developed. This framework was then checked and refined by MV and FSD before the final working framework was agreed upon. This framework was then applied to the remaining transcripts. Framework categories were arranging into distinct matrices using the ‘framework’ function in NVivo12 and then exported into Microsoft Excel. Each matrix contained summarised data for each focus group/interview (case), along with representative quotes. Initial interpretation of the data was conducted by VGW, who developed analytical themes with the domains of the TFA in mind and supplementary inductive codes. Codes and themes were generated inductively, reflecting patterns of similarity and difference within and between the focus groups and interview. Themes were subsequently reviewed and refined collaboratively (VGW, MV, FSD & DPF), thereby ensuring that the final analysis resonated with researchers from a range of disciplinary backgrounds, include those with expertise in health psychology and sociology.

## Results

### Participants

Fifty-two women participated across 10 focus groups (Black African/Black Caribbean: n = 7, low SES neighbourhoods: n = 6; n = 2, women with learning disabilities and/or autism: n = 3; n = 4, South Asian women (non-English speaking): n = 9; n = 8, women with long-term anxiety and/or depression: n = 4 and women from the Roma community: n = 3; n = 5) and one semi-structured interview (one woman with long-term anxiety and/or depression). Two focus groups were conducted with the learning disability and/or autism group, South Asian (Muslim) group and Roma group. An interview and a focus group was conducted with the long-term anxiety and/or depression group and three focus groups with the low socioeconomic group. Focus group sizes ranged from 2 to 9 women. Four focus groups were conducted with interpreters known to the women. Four support workers were present across the two focus groups conducted with women with learning disabilities and/or autism. Focus groups lasted between 70–122 minutes and the interview lasted 89 minutes. Table 1 details the demographics of the sample.

**Table 1:** Demographic and characteristics of participants (N = 52).

**Age**	
30–35	12
36–40	18
41–45	12
46–49	10
**Ethnicity**	
*Asian or Asian British: Bangladeshi*	18
*White: British*	9
*White: Roma*	8
*Asian or Asian British: Pakistani*	7
*Black African*	7
*Black Caribbean*	1
*Mixed: White and Black Caribbean*	1
*White: Other*	1
***Religion***	
*Islam/ Muslim*	25
*Christian*	21
*Hindu*	1
*No religion disclosed*	5
***First language spoken***	
*English*	19
*Bengali/Bangla*	15
*Romani/Romanian*	8
*Urdu*	6
*Chichewa*	2
*Crioulo*	1
*Twi (Ghanaian)*	1
**Index of Multiple Deprivation decile**	1-10 (M = 2.5)^a^
**Education**	
*Postgraduate qualification, i.e., Masters, PhD*	7
*Degree*	5
*‘A’ levels/other post-16 qualifications at college*	7
*‘O’ Levels/GCSEs*	18
*No qualifications*	12
*Did not answer*	3
***Employment***	
*Employed (full time)*	4
*Employed (part time)*	13
*Self-employed*	2
*Unemployed*	21
*Student*	7
*Other*	4^b^
*Did not answer*	1
***Women who have a learning disability***	7
***Women who have autism***	1
***Women with depression and/or anxiety for 1-year or more***	5
***No. of women living low socioeconomic neighbourhoods (based on postcode data)*** ^***a***^	43
***No. of women living in high socioeconomic neighbourhoods (based on postcode data)*** ^***a***^	4

**^a^** higher number meaning living in least deprived areas of the UK [31]; five women did not provide a postcode; ^b^ Includes those who detailed housewife.

Overall, all groups of women viewed offering a breast cancer risk assessment to young women aged 30–49 positively and all expressed willingness to attend if ever invited. Women’s views are presented below in the following themes: *(1) facilitating comprehension and clarity of the service, (2) anticipated accessibility of risk assessment, (3) respecting personal and cultural values in risk assessment delivery, (4) perceived confidence to engage in risk assessment and (5) balancing reassurance and worry: emotional responses to risk assessment.* Illustrative quotes are labelled with the participant’s pseudonym, along with an indicator of which focus group (FG) or interview (I) the participant took part in.

### 1. Facilitating comprehension and clarity of the service

This theme captures women’s understanding of the purpose and processes involved in a breast cancer risk assessment.

#### 1.1. Awareness as a foundation for service success.

Many women emphasised that for a breast cancer risk assessment service to be effective, women would first require greater awareness of breast cancer symptoms, risk factors and the service itself. The majority felt that breast cancer awareness within their age group was limited, particularly regarding the risk of the disease in younger women. Women also noted that official information from sources such as the NHS is not often targeted toward their age group, leaving a gap that is frequently filled by unreliable or misleading content on social media. To address this, women suggested that community-based awareness events would be essential, both to provide accurate information about breast cancer risk factors and to explain the purpose of the risk assessment service:

*…having things like today where they can put questions and they ask and they find out things is helping. So, after today they think they are more prone to take the test than they were before, without knowing what it’s about and what is implied. (Summary of discussion via interpreter, Roma-FG2)*

They suggested that such events should be tailored to specific communities and delivered in spaces where women feel comfortable and safe, such as community centres or local cultural hubs. Several women also stressed the importance of ensuring that any promotional or educational materials reflect the community being targeted, for example, using culturally appropriate language, images of women from their own backgrounds, and, where possible, having healthcare professionals from within those communities deliver sessions. This approach was seen as particularly important for engaging women from minoritised backgrounds and those with learning disabilities and/or autism, who may be less likely to access information through traditional healthcare settings.

#### 1.2. Clarity of service terminology.

A key aspect of women’s understanding of a breast cancer risk assessment service related to the terminology used to describe it. While some women felt that “breast cancer risk assessment” was clear, others, particularly those whose first language was not English, found the term confusing or anxiety-inducing. The word “risk” was perceived by some as overly harsh or suggestive of a diagnosis, while medical terminology more broadly being viewed as inaccessible. Women suggested alternative phrasing, such as “early detection test,” “breast cancer prevention test,” or even abbreviations, could help avoid misinterpretation and improve comprehension. Importantly, several women highlighted the need for simplified, non-medical language to ensure that the purpose of the service was clear to all communities:

*I think for me there’s a difference between risk assessment and cancer screening […] So, I think just explaining from that, when you say risk assessment initially, we’re thinking oh, it’s a cancer screening, we’re thinking it’s a cancer test. But it’s not, it’s how likely you are. (Esha, South Asian (Muslim)-FG1)*

#### 1.3. Clarity of the risk assessment process.

Women emphasised the importance of having clear, accessible, and step-by-step information about the process should they wish to take up the offer of breast cancer risk assessment. While some had general ideas about what the assessment might involve, many felt they would need explicit explanations of each component, including its purpose, what it would entail, and how it would benefit them personally. For example, women wanted clear guidance on what to expect depending on their risk category, including an understanding of what the care pathways could look like linked to each risk level (e.g., low, average, above-average and high):

*Amara: Are we going to have information leaflets after conducting, giving the DNA sample and whatever? Because if we can have that information explaining what each level means and what we can do that would be more helpful after the face-to-face thing. If we can have that information before we know what to expect when the results come out so we are well prepared.*

*Maya: To say when you are this level, it means that.*

*Amara: Yes, this is what it means.*

*Maya: So, you are aware okay, if they say I am at high risk this is what I can do, so that if the result is coming everyone is already calm and ready to take any results that come their way. (Low socioeconomic-FG1)*

Women also highlighted the need for accessible information about available support throughout the process, such as interpreter services, culturally appropriate support, or opportunities to ask questions. They felt that this would be particularly important for women who may face language or communication barriers. Practical strategies to aid understanding, included community events and visual resources like videos. These reflections highlight that clear, accessible communication throughout the process is essential for service coherence, ensuring women fully understand the purpose, steps, and benefits of the risk assessment service.

#### 1.4. Tailored communication of results based on risk level.

As part of their understanding of how the service should work, women reflected on how results should be communicated in a way that supports comprehension and appropriate follow-up. For those found to be at increased risk, many felt it would be essential to discuss results directly with a healthcare professional with appropriate expertise, to ensure they fully understand their risk and the early detection and prevention management options available to them:

*I probably would just need to have somebody properly explain everything to me, what it means, what I would need to implement change wise, help making the changes that we need to […] I think the whole thing for me, as long as somebody is friendly and approachable throughout and informative, tells you everything you need to know, everything that you can possibly do to change anything that they can possibly do, just arming you with all the information rather than just leaving you with loads of questions. (Leanne, low socioeconomic-FG3)*

Face-to-face (in-person or online) or telephone consultations would be preferred for those found to be at increased risk, with text messages, online portals or phone calls viewed as acceptable for arranging such appointments. Telephone and virtual consultations were particularly valued by women experiencing long-term anxiety and/or depression, as these options are seen as more accessible and less overwhelming than face-to-face appointments:

*Low risk, either just a basic letter or email is fine, but anything more than that, some level of like a discussion I think would be helpful. Again, I agree with what’s been said, not just a phone call out of the blue or anything, maybe you just set up an online appointment or online discussion or something would be fine, but something for that level of risk and what it meant for me personally, I’d appreciate it. (Bushra, anxiety and/or depression-FG1)*

By contrast, women felt that for those at low or average risk, results could be communicated by letter or text message, provided they were accompanied with breast awareness information. Yet, women suggested, regardless of risk level, all women should have the opportunity to ask questions or receive additional support if needed.

### 2. Anticipated accessibility of risk assessment

This theme captures women’s perceptions of how accessible a breast cancer risk assessment might be. As women had no prior experience of accessing a breast cancer risk assessment, they offered ideas of how the service could be made accessible and acceptable to women to reduce any anticipated burden.

#### 2.1. Primary care access and confidence in delivery.

Many women described primary care as their first point of contact for health concerns and generally trusted their GPs. However, they expressed doubts about the accessibility and reliability of a breast cancer risk assessment delivered exclusively via primary care, due to the significant barriers they encounter when securing appointments. Difficulties in securing timely consultations, experiences of having concerns dismissed, and the pressures on an already overstretched service led to questions about whether the assessment could be implemented consistently. These concerns were particularly pronounced among women from South Asian (Muslim), Black African/Caribbean (all taking part in the low socio-economic groups) and Roma backgrounds, who highlighted how language barriers, limited ability to self-advocate, and systemic inequalities could further restrict access. Women also questioned whether GPs would have the time or willingness to provide adequate follow-up, particularly for those identified as increased risk, given short consultation windows and competing professional demands:

*Jada: Would the GP be willing to work with them [secondary care to provide results to those at increased risk] to give that information as well? Because they might say that is extra work for us. I don’t know how you would be able to have that conversation with the GP for them to spare that time to speak to you…*

*Clara: Getting the appointment with the GP is another concern, because some of them you’ve got to call them up in the morning to get that slot.*

*Jada: Would the GP be willing as well to accept that from them [secondary care] because that’s extra time for them. (Low socioeconomic-FG1)*

#### 2.2. The cumulative burden of risk assessment.

When reflecting on how and where the risk assessment might be delivered, women discussed the practical efforts involved to engage, including transport access, location, and appointment availability. They noted that hospital-based assessments could present challenges due to variable transport links and favoured the idea of keeping assessments local. While there was no clear consensus on the ideal setting, with suggestions ranging from GP surgeries during annual health checks (for example those offered to those with learning disabilities), mobile breast screening units, and community or religious venues, to completing assessments privately at home, all women agreed that offering local options would help minimise anticipated burdens and encourage uptake:

*Because if somebody’s like oh, that’s just too far, that, just to see if I have a risk, they might not bother. Whereas if it is just local they might be more inclined. (Leanne, low socioeconomic-FG3)*

*So, they’re thinking somewhere local, because somebody needs to take them, like obviously if it’s in the hospital, her husband needs to take her, so the appointment time needs to be factored in, anyway. So if it’s local, she could actually go by herself. (Taslima via interpreter, South Asian (Muslim)-FG2)*

When discussing the different components of the risk assessment (e.g., questionnaire completion and saliva sampling), all groups of women agreed it would be more convenient to complete these at a single time point rather than attending multiple appointments (if in-person visits were required). Again however, there was no clear consensus on the most suitable location for completing these steps to minimise burden, with preferences varying within and across groups. For example, completion at home would reduce the burden of attending an appointment, however, some women felt that in-person interactions with a healthcare professional would be essential. With the potential for in-person appointments in mind, most women described needing maximum flexibility in order to engage, with the risk assessment needing to fit around their busy lives:

*I think everybody is available different times, so you could just give it a try and say this Monday morning or evening, and then people will tell you when they will be available. Because some people would say they’ll be available weekends, and some people aren’t, so I think all our times that we’re free vary so I cannot say okay, I’ll be free Monday to Friday as I might not be, or I cannot say weekends, some work weekends. (Maya, low socioeconomic-FG1)*

A variety of approaches for inviting women to the risk assessment and supporting questionnaire and saliva sample completion were explored, with differing views on the best strategies to encourage engagement and minimise potential burdens. Suggestions included receiving invitations and completing the questionnaire and saliva sampling by post, questionnaire completion via text messages with mobile links, or through the NHS app. Preferences for these methods varied both within and across groups, as women reflected on the personal advantages and limitations of each approach. For instance, women with long-term anxiety and/or depression highlighted that when experiencing a decline in their mental health, returning a questionnaire and saliva sample kit via post would feel especially burdensome:

*My only concern about having it sent to home is about how we’d get it back, because I am absolutely terrible at posting things. Leaving the house barrier, which seems ridiculous when it’s about posting something, it just can escalate into a bigger issue than it ever needs to be. It just happens with anxiety. (Linda, anxiety and/or depression-FG1).*

#### 2.3. Supporting cognitive engagement through simplicity and transparency.

Women consistently stressed that presenting risk assessment information in a clear, simple, and accessible way is crucial to reduce cognitive burden and enable understanding. For women with learning disabilities and/or autism, this meant using information videos and easy-read formats with straightforward text and supportive visuals:

*I think for everybody, simple text is more simple, it’s better than a complicated text do you understand. So, I think more simple things maybe that’s a good idea. (Sana, learning disabilities and/or autism-FG1)*

Similarly, women who were not fluent in English emphasised the need for translated materials and access to interpreters to ensure they could engage fully without additional mental effort. In particular, women from the Roma community highlighted the importance of keeping the pathway straightforward, emphasising that simplicity should take precedence over unnecessary complexity, which can compromise accessibility:

*…they will prefer something to be, like, even the test results and things, to be accessible to everybody who can use it to be a simple system of the results or letter or something, but to be simple words, simple system; not something so complicated. Because we try to do things sophisticated but we don’t think that sometimes it can be sophisticated but not accessible. (Summary of discussion via interpreter, Roma-FG2)*

Reflecting on the components of the risk assessment, some women recognised the importance of communicating how long the questionnaire would take to complete. Knowing this in advance was seen as essential to help them mentally prepare and manage their time, reducing the likelihood of feeling overwhelmed or disengaged during the process.

### 3. Respecting personal and cultural values in risk assessment delivery

This theme examines how a breast cancer risk assessment aligns with women’s personal values, beliefs, and cultural norms.

#### 3.1. Cultural beliefs and silence surrounding breast cancer.

For most women, conversations about breast cancer were infrequent and rarely part of everyday discussion. Unless the topic became personally relevant, such as through a loved one’s diagnosis or personal symptoms, it was generally not something openly discussed. In several communities, particularly among South Asian (Muslim), Black African/Caribbean and Roma women, breasts were described as a taboo subject, with breast cancer often surrounded by silence and stigma. A diagnosis could sometimes be viewed as an individual’s fault or as something shameful, making open discussion difficult, especially among older generations, who were seen as unlikely to share information or experiences. Some also reflected that in their communities, speaking about illness could be seen as “inviting it in”:

*So, probably it’s not necessarily that we are afraid of talking about issues like this, but it’s also in the back of our mind that we are faith people, we believe that our words have got power. We can speak things into existence, so you don’t want to speak something that you don’t want to happen to you. (Jada, low socioeconomic-FG1)*

However, many women felt these attitudes were shifting, believing younger women in their communities to be more open and willing to talk about breast health. Furthermore, the majority of women from these communities believed that by attending for a risk assessment, they could set a positive example for other women, showing them that talking about breast health and taking preventative action is not only acceptable, but important.

#### 3.2. Preserving dignity and protecting privacy.

For most women, especially those from South Asian (Muslim) backgrounds as well as those with learning disabilities and/or autism, the gender of the healthcare professional delivering the risk assessment was a significant consideration. For women with learning disabilities and/or autism, the prospect of discussing breast health or undergoing screening with a male healthcare professional was described as *‘uncomfortable’ (Michelle, learning disabilities and/or autism-FG1)*. Many South Asian (Muslim) women also expressed a strong preference for a female healthcare professional, due to cultural norms around modesty and the body being regarded as private. They also emphasised that if interpreters were required, these should ideally be female to maintain cultural sensitivity. While women understood that having a female healthcare professional for the risk assessment might not always be guaranteed (if delivered in-person), many explained that the possibility of seeing a male healthcare professional for follow-up procedures, for example for a mammogram, would make them feel uncomfortable and could deter them from attending:

*Interpreter: For breast cancer, interpreters should be a lady.*

*Researcher: Okay, what does everyone else think about that?*

*Nasrin: Yes.*

*Razia: Yes, it’s very uncomfortable talking to a guy about…*

*Priyanshi: I wouldn’t talk to a man about this. (South Asian (Muslim)-FG2)*

Although women had no significant reservations about completing a risk assessment questionnaire, some, across groups, expressed concerns about providing a saliva sample and the privacy of their DNA data. These concerns primarily centred on uncertainty about how the sample would be used beyond the risk assessment itself, reflecting broader concerns about data being shared or sold for unrelated purposes:

*…some people have funny ideas about what can be done with their DNA and where it goes and that sort of thing, so yeah, that might be something that would put some people off. (Leanne, low socioeconomic-FG3)*

To address these concerns, women emphasised the need for strong assurances that their genetic data would be used exclusively for the purpose of the risk assessment and not for any secondary reason without explicit consent. One woman also highlighted the importance of choice, suggesting that providing a saliva sample should not be mandatory and women with privacy concerns should still be able to access the service.

### 4. Perceived confidence to engage in risk assessment

This theme captures women’s confidence in their ability to engage with a breast cancer risk assessment. Since women had no prior experience with risk assessments, their reflections focused on anticipated challenges.

#### 4.1. Perceived challenges in supplying accurate risk information.

Linked to generational patterns of communication around illness and cancer diagnoses, women from South Asian (Muslim) and Black African/Caribbean backgrounds raised concerns about their ability to provide accurate family history information. Many noted that, although they may suspect a family history of breast cancer, certainty was often lacking due to older female relatives rarely disclosing their diagnoses. This led to uncertainty about how complete or reliable their family history would be, prompting concerns that their risk assessments might be less accurate:

*There is a certain taboo in the family, like, they don’t want to say what illness they’ve got, or if a person passes away, they’re not open about what condition they have passed away with, you know? So, it’s hard to retrieve that information about family members and things like that, it’s difficult. (Nasrin, South Asian (Muslim)-FG2)*

In contrast, information about health behaviours, such as smoking, alcohol intake, diet, and exercise were generally seen as easy to provide. However, some women expressed concerns about being judged, which could lead to withholding or underreporting certain behaviours, especially during face-to-face interactions with healthcare professionals. Specifically, one woman with experience of anxiety and depression recalled historically downplaying her alcohol intake due to fear of stigma linked to her mental health:

*I would talk about it but I would feel judged. Even though I know no one is judging me. Because with mental health issues and things like that, there’s some things…my history with alcohol and substance misuse, that kind of thing, I’d feel oh gosh, well, if I say how much I used to drink or whatever they might think well, of course, yes, that’s why, you’re making yourself more at risk. So I have in the past fabricated how much I’ve been drinking, and smoking as well. (Abigail, anxiety and/or depression-FG1)*

While saliva sampling was generally seen as straightforward, some women expressed concerns about their confidence in completing the process correctly if required to complete at home. To support this, they emphasised the importance of clear, step-by-step guidance, preferably in the form of a video or infographic to help them feel more assured and reduce the likelihood of making mistakes.

#### 4.2. Tailored support to facilitate engagement.

Although there was no clear consensus on the preferred method for completing a breast cancer risk assessment, it was clear that tailored support would be essential to help women feel confident in engaging with the process. Women whose first language was not English emphasised the importance of having access to interpreters throughout, from completing the questionnaire to receiving results:

*They could have interpreters ready just to help those that are maybe having difficulties with understanding whilst they’re going through the test (Jada, low socioeconomic-FG1)*

Women from the Roma community also highlighted the distinction between being able to speak and understand English conversationally and being able to read and write it fluently. To support access, they emphasised the value of having key sections of information materials, such as the most essential instructions or details translated into Romanian, helping to reduce confusion and build confidence in attending:

*…when it’s coming the invitation, if they can put in Romanian a couple of words what it is for, what will benefit and what are you going to find out with that. So they have a reason to understand. They have an understanding of the procedure, like not a very big story, I think in very simple words, but just to say what it’s for, what can benefit and what they need to do, like if they need to prepare or something for it. (Summary of discussion via interpreter, Roma-FG1)*

For women with learning disabilities and/or autism, having a trusted carer or support worker present was seen as crucial to reduce barriers and foster a sense of reassurance and confidence in taking part. One woman with learning disabilities highlighted the importance of healthcare professionals being aware of women’s individual needs in advance of the assessment. This was seen as particularly crucial for those who are non-verbal or require additional support, as such an awareness would enable a more tailored approach and help remove barriers to engagement:

*Well, they [non-verbal women] have the same feelings as us, it’s just harder for the staff because they’ve got to try and learn them more than what our staff learn us because we can speak, but someone that’s nonverbal, to give them this [the risk assessment] you’ve got to try and learn different signs and try and get [inaudible] sort of thing. (Diana, learning disabilities and/or autism-FG2).*

### 5. Balancing reassurance and worry: emotional responses to risk assessment

This theme captures women’s emotional responses to being offered a breast cancer risk assessment.

#### 5.1. Risk assessment provides hope and reassurance for the future.

All women viewed the idea of offering breast cancer risk assessments to young women positively and expressed a keen interest in attending if invited. Many highlighted the concerning prevalence of breast cancer in young women and questioned why cervical screening begins at age 25 in the UK, yet breast screening is not offered until age 50. Several women suggested that offering breast screening earlier as a result of risk assessment for those at increased risk would be a reassuring and logical step, helping to detect cancers sooner and reduce worry about developing breast cancer in the future:

*Because people younger than 50 get breast cancer all the time, and they could catch it earlier maybe if they were screening people. It’s the same with cervical cancer. If they brought the age limit down they’d catch more people. (Leanne, low socioeconomic-FG3).*

As such, women saw the opportunity for a risk assessment as a progressive step toward breast cancer prevention and early detection. Several participants emphasised that *“prevention is better than cure” (Clara, FG1, low socioeconomic),* suggesting that knowing their risk, particularly if it was high, would provide reassurance that they could access early detection and preventive measures, such as earlier screening or risk-reducing medication. Although some women recognised that receiving risk results might initially cause some worry, especially for those with mental health conditions or learning disabilities and/or autism, they felt the risk assessment would ultimately offer peace of mind, either by confirming a low-risk result or ensuring appropriate monitoring if at increased risk:

*I suppose the opportunity to know, the probability of me developing breast cancer in the future and also the follow-on from that if you’re a risk…low risk you’ve got…I mean if you’re high risk the other…the screening and the other things that are also available to confirm or rule out these things, I think that’s good. But also it gives you access to early prevention, perhaps early treatment as well and your confidence, your mental wellbeing is increased. (Asha, anxiety and/or depression-I1)*

Some women did, however, raise concerns that being provided with risk information might lead to increased anxiety which could result in excessive self-checking of their breasts. In particular, women with low-term anxiety and/or depression and those with learning disabilities and/or autism expressed that a risk assessment could *‘trigger anxieties’ (Isobel, learning disabilities and/or autism-FG2).* Ultimately though, a breast cancer risk assessment for young women was viewed as a reassuring development for the future of breast cancer prevention and early detection.

#### 5.2. Avoidance and emotional disengagement.

When reflecting on why some women in their communities might choose not to attend a breast cancer risk assessment, many women highlighted avoidance and denial as key factors. Specifically, they suggested that some women might feel too afraid to attend, fearing ‘bad’ news, while others described women they knew who would prefer not to know their risk at all. One woman with experience of anxiety suggested that, for individuals with long-term anxiety and/or depression, engagement with the service might depend on their mental state, noting that avoidance would be more likely when feeling less resilient:

*I know everything I’ve said and I would attend and everything like that, but if I’m feeling particularly unwell I think it would be too much for me to process in my head. It would potentially tip me over the edge and I wouldn’t be able to cope with that. So I’d prefer to be ignorant of it if I was really poorly. Now, I’m okay right now, I think I would want to know. But someone struggling, I think I maybe wouldn’t. (Abigail, anxiety and/or depression-FG1)*

Women from South Asian (Muslim) backgrounds also reflected on why others from their community may not attend, with feelings of denial and beliefs such as *‘it can’t happen to me’ (Razia, via interpreter, South Asian (Muslim)-FG2)* and *‘there is nothing wrong with me […] if something’s not broken, why fix it’ (Esha, South Asian (Muslim)-FG1*) and lack of family history being discussed as emotional barriers that could contribute to disengagement.

#### 5.3. The emotional weight of being ‘at risk’.

Waiting for risk results was described as a potentially stressful period, with women highlighting that being notified of an increased risk could evoke worry, panic, and fear. In particular, women with long-term anxiety and/or depression identified that an increased risk result would be hard to process and could lead them to *‘spiral’*:

*…it can be really anxiety inducing, and if you’re already quite anxious you could think oh, no, do they think that I have breast cancer, do I have it, is that certain, and if you don’t have a good support system around you, especially if you’re really struggling with your mental health at the time you might already be quite isolated. So it could trigger you to feel worse and that could potentially for some people spiral them. (Abigail, anxiety and/or depression-FG1)*

Women highlighted the need for tailored emotional support for those identified as increased risk, stressing the importance of reassuring them that being at risk is not equivalent to having breast cancer. Some women specifically noted that it should be made clear that an increased risk does not mean a woman will inevitably develop the disease.

## Discussion

Overall, all groups of women held positive views about the prospect of offering a breast cancer risk assessment to young women, viewing it as acceptable and a positive step toward prevention and early detection. Participants identified both shared and unique barriers that would need addressing before implementation, including addressing longstanding difficulties accessing GP appointments, location of services and transport links, with agreement that any service should be local, easy to reach and follow a single-appointment or ‘one-stop-clinic’ approach to reduce burden. Women across all groups valued a streamlined process with clear, transparent information at each stage, tailored to individual needs, and supported by translations of materials, interpreters, and specialist staff. Furthermore, to encourage uptake and general awareness of breast health and risk assessment, all groups supported tailored community awareness events where symptoms, risk factors, and prevention and early detection services could be discussed in a relaxed environment. For women from minoritised communities, there was a perception of breast cancer being a taboo subject, which can create a culture of silence and limit accurate sharing of family history. Additionally, for women of South Asian (Muslim) background and those with learning disabilities and/or autism, privacy and the gender of healthcare professionals were important considerations, while data privacy was a priority for all groups. Finally, while the service was seen as offering reassurance for the future, women also recognised that being identified as at increased risk could cause anxiety, particularly for those with long-term anxiety and/or depression, as well as for those with learning disabilities and/or autism.

### Relevance to existing literature

Consistent with previous literature [26,32,33], all groups of women in the present study viewed breast cancer risk assessment for young women positively and as an opportunity to offer reassurance and promote preventative action for the future. This sentiment is in line with how breast screening is viewed in general as a service that enables to the early detection of breast cancers thus providing peace of mind for those of screening age [34,35]. However, this optimism was tempered by concerns that an increased risk result could provoke anxiety and worry, particularly among women with learning disabilities and/or autism, as well as for those with long-term anxiety and/or depression. Although existing research indicates that communicating breast cancer risk results in breast screening-age population does not lead to lasting increases in cancer worry or state anxiety [36,37] these studies have not recruited women with higher levels of anxiety prior to screening, who may be more vulnerable to such concerns.

Women from ethnic minority backgrounds in the present study reported that breast cancer is still often considered a taboo subject, with a diagnosis sometimes described as ‘shameful’. These findings are consistent with literature which has explored breast cancer attitudes among different ethnic minority groups, including, South Asian breast cancer patients in the UK, Black women and women from Roma, Gypsy and Traveller communities [24,38–40]. However, this study highlights that younger women perceive this silence around breast cancer, and cancer more broadly, as generational, noting that younger women are becoming more open about discussing the disease. This shift suggests that younger women from these backgrounds may be more receptive to an invitation for risk assessment compared to older women. However, this study has shown that this generational silence may lead to uncertainty regarding family history information, indicating that for some young women gathering accurate information may be challenging, leading to concerns about the accuracy of their risk assessment.

All groups of women emphasised the value of a home-based or local, ‘one-stop-clinic’ risk assessment service to minimise the burden of attending for multiple appointments in-person. While information and support would need to be specifically tailored for different needs, all women consistently advocated for clear, transparent communication outlining what to expect at each stage of the pathway, particularly if they were found to be at increased risk. Although preferences varied regarding methods of invitation and delivery of the assessment, women from ethnic minority backgrounds in particular expressed concern about a model delivered solely through primary care, citing difficulties in securing appointments, experiences of being dismissed, and challenges in self-advocacy due to language barriers. These findings align with a systematic review of qualitative literature on patients’ experiences of communication in primary care, which highlighted that patients from ethnic minority backgrounds often report feeling rushed and stereotyped by physicians during appointments [41]. Similar findings have also been found in those which long-term health conditions including anxiety and depression where mental health stigma led to delays in cancer service referral in primary care [42]. For the women in this study, access to translation services, interpreters, and dedicated support staff was therefore seen as essential. However, ensuring effective interpretation is not always straightforward, with Bayliss et al [43] noting that interpreters may not fully understand a patient’s specific dialect.

In line with previous research highlighting limited awareness of breast cancer risk and self-checking among younger women [44,45], all groups of women further emphasised that the effectiveness of a risk assessment service would depend on improving baseline awareness of breast cancer symptoms, risk factors, and the service itself. Some women reported that some might not attend a risk assessment if they had no known family history, assuming the service was not relevant to them, a misconception also reflected in the literature, where a lack of family history is often viewed as protective despite other risk factors [46]. To address this, participants advocated for community-based awareness initiatives that offer accessible and culturally tailored information in trusted settings. This approach reflects the UK Department of Health and Social Care’s long-term plan to bring healthcare closer to communities [47], as well as with research highlighting healthcare professionals’ support for more proactive, community-based risk assessment services [48]. It also aligns with evidence that culturally relevant materials, delivered by trusted community representatives, can enhance understanding and engagement with screening, particularly among minoritised groups [49–51]. However, few breast cancer awareness interventions currently exist for women with learning disabilities [52].

### Strengths and limitations

A key strength of this study was its success in engaging a broad and diverse range of participants, including women from different minoritised communities, socio-economic neighbourhoods, and with varied health and support needs. This diversity provided rich insights into how a breast cancer risk assessment service for women aged 30–49 might be understood and accessed across different communities from groups typically under-represented in research. However, this study was not without its limitations. Breast screening non-attendance is higher among women living in lower socio-economic neighbourhoods [16,18,19]. Although White-British women make up the largest proportion of the UK population invited for breast screening, there is limited evidence to suggest they also represent the largest group of non-attenders. Despite efforts to recruit White-British women from lower socio-economic backgrounds, they were under-represented in the present sample. In addition, the findings may be subject to participation bias, as women who chose to take part may have had a greater pre-existing interest in breast cancer and breast health than those who did not participate. This could have contributed to higher levels of acceptability of, and interest in, breast cancer risk assessment than may be observed in the wider population. Also, women who may face additional needs due to physical disabilities or those who may experience weight stigma were not deliberately recruited for this study.

While the focus group format was effective for most participants, it may not have been the most suitable method for eliciting in-depth perspectives from women in the Roma community and those with learning disabilities and/or autism. Greater time for rapport-building and more interactive, adapted approaches may have enabled fuller engagement and richer contributions.

### Research team reflexivity statement

The multidisciplinary research team had expertise spanning clinical practice, healthcare, qualitative methods, applied health research, and breast cancer risk research. The focus groups and interview were conducted by VGW, HM, LMcW, MV and FSD. VGW, LMcW HM, and MV are White women, FSD is a Latin American woman. These researchers all have extensive experience in qualitative research methods. VGW and LMcW have particular expertise in breast cancer risk research and engaging under-served and minoritised communities in health research. HM has experience working in applied healthcare research with individuals with mental and physical health conditions in community and clinical settings. MV, LMcW and FSD have experience working with individuals with chronic conditions, including a range of mental health conditions.

As researchers, we could largely be considered outsiders to the communities involved in this study. To address this, the team worked closely with community gatekeepers, charities, and local organisations throughout the research process to build trust, strengthen community understanding, and support meaningful engagement. However, for the mental health group, there was no opportunity to engage with relevant charities during the planning stage. As the researcher who conducted the focus group and interview (MV) had previous professional experience working with young people and individuals living with chronic conditions, this experience informed their approach to facilitating discussions with participants. In particular, the researcher was mindful of avoiding judgemental responses to discussions of lifestyle habits (e.g., alcohol consumption), using sensitive and non-stigmatising language, and avoiding potentially loaded terms such as *illness* where appropriate.

The research teams’ positionality may have also influenced how women’s experiences and perspectives were interpreted during data collection and analysis, with the potential for some cultural or contextual nuances to be overlooked or misinterpreted. To enhance the credibility of the findings, the lead researchers’ interpretations were discussed and reviewed across the multidisciplinary research team throughout the analytic process, enabling reflexive consideration of alternative explanations and perspectives. In addition, preliminary findings were shared with women involved in the subsequent phase of the research, who reported that the findings resonated with their experiences and views.

### Implications for practice

As breast cancer risk assessment for young women is now being trialled in the UK [11–13], it is increasingly important to consider how such a service is organised to ensure procedures are appropriate and inclusive for all groups of women. The present study showed that there was no single preferred method of contacting women about breast cancer risk assessment, as preferences were highly individual. When trialling a risk assessment for under-served communities, it will be important to record women’s preferred methods of communication to ensure messages are delivered in a way that is accessible and appropriate for them. In addition, information on support needs, such as translation services, interpreters, or easy read/visual formats, should be collected so that communication about the service is tailored and inclusive for all.

When implementing breast cancer risk assessment for young women, it will also be important to address women’s concerns about privacy and the handling of DNA data collected from saliva samples. Clear communication about how genetic information will be stored, protected, and used would likely help build trust and confidence in the service. In addition, some women in the present study expressed a preference for female healthcare professionals, highlighting the need for flexibility in workforce planning to ensure that care is both sensitive and responsive to individual needs.

Finally, all women valued the importance of community awareness and outreach events to promote breast cancer risk assessment. A risk assessment trial in this area should embed strategies for community engagement, making use of communication channels that are most relevant to different groups. Tailoring these initiatives to reflect cultural and community needs could help build trust, improve confidence, and support greater uptake by showing why the service is meaningful and relevant to the community.

### Future research

Future research should use co-design approaches with women from diverse under-served groups to develop a breast cancer risk assessment service that reflects both shared and community-specific needs. These tailored interventions could then be tested in clinical settings to evaluate whether they improve uptake compared to standard, non-targeted initiatives. Furthermore, given women’s preference for local, community-based assessments, future trials should incorporate a community-based arm, to enhance accessibility and engagement. Finally, future research should consider engaging women from under-served groups who have not been included here. For example, White women from low socioeconomic neighbourhoods represent a large group who often do not attend screening, thus further work is needed to explore the reasons for this, including the impact of comorbidities and other competing demands on their health and wellbeing.

## Conclusion

In conclusion, all groups of women found the offer of a breast cancer risk assessment for women aged 30–49 acceptable, but successful implementation would depend on ensuring the service is inclusive, culturally sensitive, and responsive to diverse needs. Women highlighted the importance of community-based awareness initiatives, clear and tailored communication, privacy and data security assurances, and flexible care pathways that minimise practical barriers to access and is seen as reassuring. Designing a proactive service with these needs in mind may strengthen trust, enhance uptake, and reduce inequalities, ensuring breast cancer risk assessment for young women is both accessible and empowering.

## Supporting information

S1 File Interview topic guides. (DOCX)
