# Supplementary material for: Acceptability of breast cancer risk assessment for women aged 30–49: Views of women from six under-served groups
Source: PLoS One. 2026 Aug 17;21(8):e0356263. doi: 10.1371/journal.pone.0356263 (PMC13480479; doi:10.1371/journal.pone.0356263)
Supplement: Supplementary file 1 [file pone.0356263.s001.docx]

**S1 Appendix**

**Topic guide – low SES, long-term A/D and SA(Muslim) groups**

**Introducing a breast cancer risk assessment into the NHS for women under 50 years of age: a focus group study**

***For the ethics committee and researchers:*** *this study is about exploring young women’s (30-49 years of age) views and opinions on the introduction of a breast cancer risk assessment into the NHS. These women are from backgrounds that usually would not attend for screening or who experience health inequalities when accessing services in the NHS. Therefore the focus groups (or interviews) will focus on three main areas: (i) women’s understanding of cancer risk and attitudes towards cancer risk assessment, (ii) women’s thoughts about a breast cancer risk assessment including what would be appealing about such a service and what could affect attendance and (iii) women’s perceived barriers and facilitators for attending additional breast screening.*

**Introduction to the topic**

***For the researcher following consent but prior to starting the focus group/interview:*** Thank you for agreeing to take part in this focus group/interview today. As you know in this focus group/interview we will be discussing your thoughts on a breast cancer risk assessment for young women aged between 30 and 49 years of age. In this focus group/interview we want to find out what you would think about this and what might be good about this service as well as what issues you could face. We will first start by talking about cancer risk in general and then we will move on to talk more about the idea of a breast cancer risk assessment for young women in the NHS.

The focus group/interview may last up to two hours. If at any time you would like to take a break please do let one of us know. This is not a test. There are no right or wrong answers, we just want to hear what you have to say. You do not have to answer any questions that you don’t want to. As you know we will be recording the focus group/interview. This is so we can listen to you all without taking notes and we can portray your contributions accurately. We may ask you to explain things further in the focus group/interview. This is not because you are not being clear, but when we are presenting this work we need to make sure it is in your own words and not ours. We will also be taking notes throughout and we will feed these back to you at the end.

***Focus group only:*** Finally during the focus group it is important to listen to everyone’s opinions and try not to interrupt or talk over one another. Someone might have a different opinion and that is ok, as all opinions are valid and to be respected.

Does anyone have any questions before we start?

**Focus group/interview questions and topic areas**

***Breast cancer and breast cancer risk***

1. Let’s start off by thinking about breast cancer in general. In the community or culture you are from, how do people usually talk about breast cancer?
   a. What do you think women like you think when they hear the term ‘breast cancer’?
   b. Is it spoken about much?
   c. Is it important to women like you?
2. Is being at risk of breast cancer something that is considered in your community/culture?
   a. Is thinking about breast cancer risk important?
   b. Is it as important as other illnesses and diseases?
3. If you are comfortable in sharing, have you ever talked to your GP about breast cancer or breast cancer risk?
   a. If not, why do you think that is?

***Cancer risk assessments (understanding & attitudes):***

4. Let’s talk now about what a cancer risk assessment might be. Does anyone have any ideas of what this might entail (don’t worry this isn’t a test)?
   a. What would you think if you received a letter from the NHS inviting you for a cancer risk assessment?
   b. What do you think might happen during a cancer risk assessment / what might it tell you?
5. Would a cancer risk assessment be something you’d want to have? Why/why not?
   a. What would be appealing about a cancer risk assessment?
   b. What concerns do you think you’d have about a cancer risk assessment?
   c. Do you think it is important to know your own risk of developing cancer? Why/why not?
6. What do you think about the service being called a ‘cancer risk assessment?
   a. Is this description acceptable to you? (Why/why not?)
   b. Do you think it needs to be called something different or is this ok? (Why?)

*[note to researcher – may need to explain what a cancer risk assessment is prior to the next line of questioning so that women are more informed when answering].*

***Women’s thoughts about a breast cancer risk assessment:***

*Let’s move on now to talk specifically about a breast cancer risk assessment for women your age.*

7. What would you think if you were offered a breast cancer risk assessment at your current age?
   a. Is knowing your breast cancer risk important to you in this stage of your life?
   b. How do you think you should be invited for a breast cancer risk assessment [prompts: by the GP, letter, text message, email, mobile app?], and why?

*A woman’s breast cancer risk is calculated using different risk factors. Such as whether women have a family history of breast cancer, reproductive (no. of children) and hormonal factors (age at period/menopause), genetic factors and health behaviours (i.e. diet, alcohol intake, smoking).*

8. How would you feel about giving information about your family history?
   a. Would you face any issues or challenges in gathering this information? [i.e. questions about FH could be quite detailed].
9. How would you feel about giving information about your health behaviours (i.e. diet, exercise, alcohol intake, smoking?
   a. Would there be any information that would be difficult to provide?
10. How would you like to be asked questions about yourself for the breast cancer risk assessment? [Prompts: via a letter and send back, online, helpline, in-person at a GP appointment, in-person at a community setting, at existing health check appointments, other?].
    a. These questions could be asked via a questionnaire. What do you think about that?
       i. Could there be any challenges to completing this questionnaire?
11. How would you feel about giving a saliva sample so that HCPs can look at your DNA/genetics to calculate your breast cancer risk?
    a. Is this ok? Would you have any concerns?
12. Where do you think this saliva sample should be taken? [Prompts: at home, at GP, community setting, also by whom (self/GP/nurse)].
    a. Again do you think you could face any challenges to giving this sample?

*After all this information is collected HCPs will calculate a breast cancer risk score for women. Women can be placed in one of four categories: low risk, average risk, above-average risk or high risk.*

13. What do you think about the labels we have given to these categories?
14. How do you think women should be told their breast cancer risk results? [Prompts: letter, email, text, in person (by whom)].
    a. Why/why don’t you think that would be a good approach?
15. Should all risk types be told their results in the same way?

***Women’s perceived barriers and facilitators for attending additional breast screening***

*Young women who are told that they are at above-average or high risk of breast cancer could be offered breast screening early. This is so that any cancers can be detected and caught early, reducing the need for aggressive treatments and improving their prognosis.*

16. If you are comfortable in sharing, do you plan on attending breast screening when you are eligible at age 50? [why/why not?]
17. Where do you think screening should take place for young women found to be at above-average or high risk and why?
    a. What challenges could young women like you face for attending screening if they were found to be at above-average or high risk? [Prompts: Location? Accessibility concerns? Transport? Time? Emotional barriers? Cultural?].
    b. What would help young women attend screening appointments?
       i. Reminders? How?
       ii. Accessible location? Where?
       i. Accessible tailored service?
       ii. Emotional support?
       iii. Appointment flexibility?

***Support: how, where and who from?***

18. What support do you think women at above-average and high risk of breast cancer might need?
    a. Where do you think the best place would be to access support or further care? [prompts: hotline/websites/GP/Primary Care; Secondary/Specialist Care?]
    b. What support do you think you would specifically need if you were at above-average or high risk?
19. What support do you think women at low or average risk of breast cancer might need?

*Some women might not want to take up the offer of a breast cancer risk assessment.*

20. From your point of view, why might women from your community or culture not want to have a breast cancer risk assessment?
    a. i.e. What might affect women choosing to have a risk assessment (What could be done about this?)
21. What might you/your community or culture *need* in order to make accessing a breast cancer risk assessment easy?
    a. What would encourage you to have a breast cancer risk assessment?

***Overall thoughts***

22. Overall, thinking about the breast cancer risk assessment as a whole:
    a. What do you think is good about it?
    b. Would you have any concerns? I.e. what would be off putting?
23. What is the most important thing that you think we should take away from this discussion today?

**Finishing comments**

Thank you all for your time today. We really appreciate it.

- Is there anything else anyone would like to add?
- Is there anything you think we would have covered and we haven’t?
- Do you have any questions for us?

Thank you for taking part today, we hope you found what we talked about interesting. The recording of this focus group/interview will now be typed up but all identifiable information will be replaced and we will replace your name with a fake name. If you have any questions after today then please do not hesitate to contact me or the team. Our contact details are on the information sheet we gave you and on the debrief sheet we will now give out which also contains useful resources you can access if you want to know more about breast cancer and risk. If you would like a copy of the findings once they have been finalised, I would be happy to send them to you. Just let me know. Again thank you very much for today and the contributions you have made. It really is invaluable.

**Topic guide – Roma community**

**Introducing a breast cancer risk assessment into the NHS for women under 50 years of age: a focus group study**

***For the ethics committee and researchers:*** *this study is about exploring young women’s (30-49 years of age) views and opinions on the introduction of a breast cancer risk assessment into the NHS. These women are from backgrounds that usually would not attend for screening or who experience health inequalities when accessing services in the NHS. Therefore, the focus groups (or interviews) will focus on three main areas: (i) women’s understanding of cancer risk and attitudes towards cancer risk assessment, (ii) women’s thoughts about a breast cancer risk assessment including what would be appealing about such a service and what could affect attendance and (iii) women’s perceived barriers and facilitators for attending additional breast screening.*

**Introduction to the topic**

***For the researcher following consent but prior to starting the focus group/interview*:** Thank you for agreeing to join this focus group (or interview) today. We will be talking about breast cancer and what you think to young women being offered something called a breast cancer risk assessment. This service would give young women an idea of how likely they are to develop breast cancer. We want to hear your thoughts about this and what you think might be good or not so good about it.

First, we will talk about cancer risk in general, and then we will talk more about how we can check breast cancer risk for women aged 30 to 49 in the NHS.

The focus group might last up to two hours. [if interview: The interview might last up to one hour]. If you need a break at any time, please let us know. We have some questions to help us with our discussion today. There is no right or wrong answer here. We just want to hear what you think. You don’t have to answer any question you don’t want to. If a question does not make sense, please let me know and I can find another way to ask.

As you know, we will be recording the focus group (or interview). This is so we can listen to what you say and make sure we understand you correctly. We might ask you to explain something again. This is not because you are being unclear, but because we want to capture what you said in your own words. We will also take some notes and can show them to you at the end.

***Focus group only:*** It’s important that everyone listens to each other and does not interrupt or talk over someone else. It is okay if someone has a different opinion. All opinions are important and should be respected. What you or other people say today is private and we should not repeat what anybody says when we have finished today.

Does anyone have any questions before we start?

[Starter activity – lets go round the group and introduce ourselves and share one thing that made us smile today *(note to researcher – this will help the transcriber identify voices)*].

**Focus group/interview questions and topic areas**

We know that the subject of breast cancer be quite scary to talk about, but it is important that know what the signs might be and act early so that we can identify it early and lead happy healthy lives. In the UK 1 in every 7 women will be diagnosed with breast cancer, that is about 57,000 cases a year. But the positive thing is that 76% of women diagnosed will make a full recovery. **What do you think about that?**

***General: breast cancer & health***

- **Have you heard about breast cancer before?**

(if yes) – would you like to share what you know about breast cancer?

(if no) – that’s okay, I can tell you. Breast cancer is when cells in the breast grow in a way that they shouldn’t. **Would anyone like to ask questions?**

- **Has anyone talked to you about breast cancer before?**

(if yes) – what was that like? Can you remember what they told you?

- **Have you ever spoken to friends or family about breast cancer?**

(if yes) – if you are happy to share, what did you talk about?

(if no) – why do you think that is?

- **If you were worried about breast cancer who would you trust to talk to?**

(Prompts: support staff, friends, family, GP, nurse).

***Symptoms and causes of breast cancer***

- **Would anyone like to share what they think might be a symptom or a sign of breast cancer?**

*(lump in breast/chest/armpit, pushed in nipple, persistent pain, skin changes, changes in shape, fluid leaking from breast – show images (paper/PowerPoint))*

- **Have you ever checked your breasts for changes before?**

(Example – some people check their breasts in the shower, some check in front of the mirror).

(Follow-up – has a doctor or nurse ever talked to you about how to check your breasts?)

- **Would anyone like to share what they think might cause breast cancer?**

*(age, family member has had BC, diet and lifestyle behaviours, weight, age at first period, if you have had children, your genes and DNA – show images (paper/PowerPoint)).*

***Breast cancer risk assessment***

Researchers at the University of Manchester/Cambridge and in the NHS are thinking about asking young women if they would want to know how likely they are to get breast cancer in the next 10-years. We think this is a good idea because more women between the ages of 30-49 are getting breast cancer and sometimes the breast cancer they get can be very serious.

- **Do you think it would be a good thing or a bad thing for women in your community to know their breast cancer risk? Why?**
- **How do you think knowing this information could affect women’s lives?**
- **How do you think women would feel if they knew how likely they were to get breast cancer?** (Bad, good, informed, worried, upset, scared?

***Risk assessment process***

To find out how likely women are to get breast cancer, women could be offered a breast cancer risk assessment.

- **Would you want to have a risk assessment?**
- **What would you need to know about the assessment?**
- **How do you think women would like to be invited to a breast cancer risk assessment if they wanted one?**
  Invite in the post?
  Email?
  NHS App?
  Via a doctor or nurse at a GP surgery?
- **What is the best way for people to share health information with your community?**

(Prompt: “Would pictures help? Do you like videos, leaflets, or someone explaining things in person?”)

If women wanted to find out how likely they were to get breast cancer they could be asked to fill in a questionnaire. This might ask women questions about, (i) if they have any family members with BC, (ii) if they have children, how old they were when they had them, (iii) diet and lifestyle questions (smoking, alcohol intake, exercise) and (iv) how old they were when they started their period.

- **Would it be easy or hard to answer these types of questions? (Why/why not?)**
- **Should the questions come in the:**
- Post?
- By email?
- Be asked over the telephone?
- Be done face to face? (with who/where)?
- **What support might women in your community need to answer the questions?**
- Family member?
- Doctor? Nurse?
- Who would you trust to help?

After women have answered the questions, they might be asked to swab their gums so a sample of spit can be taken. **(explain like the covid test).** This is so doctors can look at things in the DNA **(things in our bodies that make us who we are and unique)** that might increase the likelihood of getting breast cancer.

- **Do you think women in your community would want to do a swab of their gums?**
- **Why/why not?**
- **What could be good about it? What could be bad?**
- **Where do you think women would want to do this?** [prompts: at home, at a GP surgery, in a community centre?]
- **Do you think women could do this on their own or would they need support?** [From who?]

***Risk assessment results***

So with all the things we have talked about, the doctors would put all this together and women would get a result telling them how likely they are to get breast cancer.

- **How do you usually like to be told about your test results?**
- By post?
- By email?
- Over the telephone?
- In person?
- Who do you like to be told by?
- **What do you think it means when we say someone has an ‘increased risk’ of getting breast cancer?**

(If needed, explain: “It means they might be more likely to get it, but it doesn’t mean they will.”)

- **How might women feel if they were told they were at an ‘increased risk’ of getting breast cancer?** (prompts: worried, scared, happy to know?).
- **How might women feel if they were told they were at a ‘low risk’ of getting breast cancer?** (prompts: would that be good?)

A woman at increased risk could be offered what we call a mammogram or breast screening.

- **Does anyone know what a mammogram or breast screening is?** [researcher explain if not].
- **Do you think women in your community would want to go to breast screening if they were invited? Why/why not?**
- **What do you think they would want to know?**
- **What support do you think women would you need?**
- ***Overall thoughts***
- **What is the most important thing you think we should take away from our discussion today?**
- **What gives you hope about the discussions we have had today?**

**Finishing comments**

Thank you all for your time today. We really appreciate it.

- Is there anything else anyone would like to add?
- [note taking researcher] is there anything you would like to ask the group?
- Do you have any questions for us?

Thank you for taking part today. We hope you found the discussion interesting. We will now type up the recording from the focus group/interview, but we will replace any information that could identify you, including your name, with a fake name. We will use the notes we took today to help but then we will destroy these when we have finished the project.

If you have any questions after today, please feel free to contact me or the team. Our contact details are on the information sheet we gave you and on the debrief sheet we will now give out together with useful resources you can access if you want to know more about breast cancer and risk. If you would like a copy of the results when they are finished, I’d be happy to send it to you. Just let me know.

Thank you again for your time and for sharing your thoughts. Your help is really important to us.

**Topic guide – LD/A community**

**Introducing a breast cancer risk assessment into the NHS for women under 50 years of age: a focus group study**

***For the ethics committee and researchers:*** *this study is about exploring young women’s (30-49 years of age) views and opinions on the introduction of a breast cancer risk assessment into the NHS. These women are from backgrounds that usually would not attend for screening or who experience health inequalities when accessing services in the NHS. Therefore, the focus groups (or interviews) will focus on three main areas: (i) women’s understanding of cancer risk and attitudes towards cancer risk assessment, (ii) women’s thoughts about a breast cancer risk assessment including what would be appealing about such a service and what could affect attendance and (iii) women’s perceived barriers and facilitators for attending additional breast screening.*

**Introduction to the topic**

***For the researcher following consent but prior to starting the focus group/interview*:** Thank you for agreeing to join this focus group (or interview) today. We will be talking about breast cancer and what you think to young women being offered something called a breast cancer risk assessment. This service would give young women an idea of how likely they are to develop breast cancer. We want to hear your thoughts about this and what you think might be good or not so good about it.

First, we will talk about cancer risk in general, and then we will talk more about how we can check breast cancer risk for women aged 30 to 49 in the NHS.

The focus group might last up to two hours. [if interview: The interview might last up to one hour]. If you need a break at any time, please let us know. We have some questions to help us with our discussion today. There is no right or wrong answer here. We just want to hear what you think. You don’t have to answer any question you don’t want to. If a question does not make sense, please let me know and I can find another way to ask.

As you know, we will be recording the focus group (or interview). This is so we can listen to what you say and make sure we understand you correctly. We might ask you to explain something again. This is not because you are being unclear, but because we want to capture what you said in your own words. We will also take some notes and can show them to you at the end.

***Focus group only:*** It’s important that everyone listens to each other and does not interrupt or talk over someone else. It is okay if someone has a different opinion. All opinions are important and should be respected. What you or other people say today is private and we should not repeat what anybody says when we have finished today.

Does anyone have any questions before we start?

[Starter activity – lets go round the group and introduce ourselves and share one thing that made us smile today *(note to researcher – this will help the transcriber identify voices)*].

**Focus group/interview questions and topic areas**

Before we start chatting about breast cancer, **would anyone like to share why they wanted to come a long today and take part?**

***General: breast cancer & health***

1. **Have you heard about breast cancer before?**

(if yes) – would you like to share what you know about breast cancer?

(if no) – that’s okay, I can tell you. Breast cancer is when cells in the breast grow in a way that they shouldn’t. **Would anyone like to ask questions?**

2. **Has anyone talked to you about breast cancer before?**

(if yes) – what was that like? Can you remember what they told you?

3. **Have you ever spoken to friends or family about breast cancer?**

(if yes) – if you are happy to share, what did you talk about?

4. **Have you ever thought about how likely you are to get breast cancer?**
5. **If you were worried about breast cancer who would you trust to talk to?**

(Prompts: support staff, friends, family, GP, nurse).

***Symptoms and causes of breast cancer***

6. **Would anyone like to share what they think might be a symptom or a sign of breast cancer?**

*(lump in breast/chest/armpit, pushed in nipple, persistent pain, skin changes, changes in shape, fluid leaking from breast)*

7. **Have you ever checked your breasts for changes before?**

(Example – some people check their breasts in the shower, some check in front of the mirror).

(Follow-up – has a doctor or nurse ever talked to you about how to check your breasts?)

8. **Would anyone like to share what they think might cause breast cancer?**

*(age, family member has had BC, diet and lifestyle behaviours, weight, age at first period, if you have had children, your genes and DNA).*

***Breast cancer risk assessment***

Researchers at the University and in the NHS are thinking about asking young women if they would want to know how likely they are to get breast cancer in the next 10-years.

9. **Would you want to know how likely you were to get breast cancer in the next 10-years?**

(Red, orange, green cards – they can indicate how they feel and have a discussion).

10. **Why do you think some women might not want to know this?**

(Scared? Worried? Not important?)

11. **What would be good about knowing how likely you were to get breast cancer?**
    a. **What would be bad about it?**

(Prompts: would have information, would know what they could do? worried, scared?)

12. **How do you think you would feel if you knew how likely you were to get breast cancer?**

(bad, good, informed, worried, upset, scared?

***Risk assessment process***

To find out how likely you are to get breast cancer, women could be offered a breast cancer risk assessment.

13. **Would you want to have an assessment?**
    a. **What would you need to know about the assessment?**
14. **How would you like to be invited to a breast cancer risk assessment if you wanted one?**
    a. Invite in the post?
    b. Via a doctor or nurse at a GP surgery?
15. **What is the best way for people to share health information with you?**

(Prompt: “Would pictures help? Do you like videos, leaflets, or someone explaining things in person?”)

If you wanted to find out how likely you were to get breast cancer you could be asked to fill in a questionnaire first of all. This might ask you questions about, (i) if you have any family members with BC, (ii) if you have children, how old you were when you had them, (iii) diet and lifestyle questions (smoking, alcohol intake, exercise) and (iv) how old you were when you started your period.

16. **Would it be easy or hard to answer these types of questions? (Why/why not?)**
17. **Should the questions come in the:**
    a. Post?
    b. By email?
    c. Be asked over the telephone?
    d. Be done face to face? (with who/where)?
18. **What support might you need to answer the questions?**
    a. Support worker? Family member?
    b. Doctor? Nurse?
    c. Who would you trust to help?
19. **Could this be done at the annual health check?**
    a. **Or by a learning disability nurse?**

After women have answered the questions, they might be asked to swab their gums so a sample of spit can be taken. **(explain like the covid test).** This is so doctors can look at things in the DNA **(things in our bodies that make us who we are and unique)** that might increase the likelihood of getting breast cancer.

20. **Would you want to do a swab of your gums?**

(Red, orange, green cards – they can indicate how they feel and have a discussion).

21. **What could be good about it? What could be bad?**
22. **Where do you think you would want to do this?** [prompts: at home, at a GP surgery, in a community centre?]
23. **Could you do this on your own or would you want support?** [From who?]
    a. **Could it be done at an annual health check?**
    b. **By a learning disability nurse?**

***Risk assessment results***

So with all the things we have talked about, the doctors would put all this together and you would get a result telling you how likely you are to get breast cancer.

24. **How do you usually like to be told about your test results?**
    a. By post?
    b. By email?
    c. Over the telephone?
    d. In person?
    e. Who do you like to be told by?
25. **What do you think it means when we say someone has an ‘increased risk’ of getting breast cancer?**

(If needed, explain: “It means they might be more likely to get it, but it doesn’t mean they will.”)

26. **How might you feel if you were told you were at an ‘increased risk’ of getting breast cancer?** (prompts: worried, scared, happy to know?).
27. **How might you feel if you were told you were at a ‘low risk’ of getting breast cancer?** (prompts: would that be good?)

A woman at increased risk could be offered what we call a mammogram or breast screening.

28. **Does anyone know what a mammogram or breast screening is?** [researcher explain if not].
29. **Would you want to go to breast screening if you were invited? Why/why not?**
    a. **What would you want to know?**
    b. **What support would you need?**

***Overall thoughts***

30. **What is the most important think you think I should take away from our discussion today?**

**Finishing comments**

Thank you all for your time today. We really appreciate it.

- Is there anything else anyone would like to add?
- [note taking researcher] is there anything you would like to ask the group?
- Do you have any questions for us?

Thank you for taking part today. We hope you found the discussion interesting. We will now type up the recording from the focus group/interview, but we will replace any information that could identify you, including your name, with a fake name. We will use the notes we took today to help but then we will destroy these when we have finished the project.

If you have any questions after today, please feel free to contact me or the team. Our contact details are on the information sheet we gave you and on the debrief sheet we will now give out together with useful resources you can access if you want to know more about breast cancer and risk. If you would like a copy of the results when they are finished, I’d be happy to send it to you. Just let me know.

Thank you again for your time and for sharing your thoughts. Your help is really important to us.
