# Supplementary material for: Acceptability of breast cancer risk assessment for women aged 30–49: Views of women from six under-served groups
Source: PLoS One. 2026 Aug 17;21(8):e0356263. doi: 10.1371/journal.pone.0356263 (PMC13480479; doi:10.1371/journal.pone.0356263)
Supplement: Supplementary file 2 [file pone.0356263.s003.docx]

|  | **Reviewer and editorial comments** | **Response** | **Location of change** |
| --- | --- | --- | --- |
| **Editor comments** | Please ensure that your manuscript meets PLOS ONE's style requirements, including those for file naming. | *Thank you for this comment. Looking at the guidance we believe that the manuscript is now formatted in PLOS One’s style.* | *Throughout manuscript.* |
|  | Please state what role the funders took in the study. If the funders had no role, please state: "The funders had no role in study design, data collection and analysis, decision to publish, or preparation of the manuscript."  If this statement is not correct you must amend it as needed.  Please include this amended Role of Funder statement in your cover letter; we will change the online submission form on your behalf. | *Thank you for this comment. We have included the suggested statement to the cover letter:*  *This study was funded by the Cancer Research UK International Early Detection of Cancer Alliance (ACED) (EDDAMC-2023/100004) and the Manchester Academic Health Science Centre (MAHSC): Cancer Domain. DPF, LMW and HM are supported by the National Institute for Health Research Manchester Biomedical Research Centre (NIHR203308). DF is a NIHR Senior Investigator (NIHR305827). LMW is supported by a Cancer Research UK Career Development Fellowship (RCCCDF-May25/100006). The views expressed are those of the author(s) and not necessarily those of the NIHR or the Department of Health and Social Care. The funders had no role in study design, data collection and analysis, decision to publish, or preparation of the manuscript.* | *Cover letter* |
|  | In the online submission form, you indicated that your data is available only on request from a third party. Please note that your Data Availability Statement is currently missing the name of the third party contact or institution / contact details for the third party, such as an email address or a link to where data requests can be made. Please update your statement with the missing information. | Thank you for this comment. We have updated the Data Availability Statement. The data is now available via FigShare at the University of Manchester  <https://doi.org/10.48420/32743704>  We are now clear that these data are free to upload, and do not require permissions. |  |
|  | Please include captions for your Supporting Information files at the end of your manuscript, and update any in-text citations to match accordingly. | *Thank you for this comment. We have one supporting file and this now has been labelled S1 in the manuscript and the following has been added to the end of the manuscript:*  *Supporting Information*  *S1 File. Interview topic guides* | *Pg. 9-10, lines 184, 192, 204.*  *Pg. 48, lines 1045-1046* |
|  | If the reviewer comments include a recommendation to cite specific previously published works, please review and evaluate these publications to determine whether they are relevant and should be cited. There is no requirement to cite these works unless the editor has indicated otherwise. | *Thank you this reminder. Reviewer 1 has suggested a paper to read but not to be cited.* | *NA* |
|  | Please review your reference list to ensure that it is complete and correct. If you have cited papers that have been retracted, please include the rationale for doing so in the manuscript text, or remove these references and replace them with relevant current references. Any changes to the reference list should be mentioned in the rebuttal letter that accompanies your revised manuscript. If you need to cite a retracted article, indicate the article’s retracted status in the References list and also include a citation and full reference for the retraction notice. | *Thank you for this comment we have checked all the references and made changes in line with the formatting guidance.* | *Pgs. 41-48.* |
| **Reviewer 1** | Woof and colleagues’ study provides important insights on the acceptability of breast cancer risk assessment for women aged 30–49 among women from different under-served groups. It is superbly written, with very clear methods, results, and a great discussion. The study is of very high quality and was a pleasure to review.  So I only have very minor comments, listed below. | *Thank you for providing such positive feedback. We have so pleased that you appraised the manuscript as high quality and you enjoyed reviewing our research.*  *Thank you also for providing comments to improve the manuscript. We hope we have managed to address each one sufficiently.* | *NA* |
|  | Lines 244-248: While acknowledging intersectionality, it would be helpful to indicate the number of participants in each of the focus groups. | *Thank you this comment we have provided more information on this:*  *Fifty-two women participated across 10 focus groups (Black African/Black Caribbean: n=7, low SES neighbourhoods: n=6; n=2, women with learning disabilities and/or autism: n=3; n=4, South Asian women (non-English speaking): n=9; n=8, women with long-term anxiety and/or depression: n=4 and women from the Roma community: n=3; n=5) and one semi-structured interview (one woman with long-term anxiety and/or depression).*  *We have also identified an error in our reporting of the groups and have fixed this also in the title, abstract and throughout the text.* | *Pg. 13, lines 281-285.* |
|  | Table 1: for clarity, it would be helpful to provide the total numbers of participants who matched to each of the five communities/background listed in the Methods (lines 108-115) in this table, noting some participants may be part of multiple groups. These numbers depend on the definition of some of these groups, so are currently not fully clear from the table. | *Thank you for this comment.*  *We have provided extra information in the table, specifically to do with socioeconomic status as information on ethnicity, learning disabilities and mental health status was already provided.*  *However, we think that given the multiple groups and degree of intersectionality (where a person may be black, living in a low SES area and have depression), to present a detailed quantitative breakdown of intersectionality would be complex and difficult to understand. Further, it may be misleading, as we did not, e.g. ask women recruited on the basis of where they lived or their ethnic group whether they were experiencing mental health issues.*  *We hope with this information and the information provided to address the previous comment that the intersectionality of the groups is clearer.* | *Pg. 13-15* |
|  | Strengths and limitations: it may be worth noting that the results could be subject to some participation bias, in that those who chose to participate in the study could have stronger general interest in breast cancer, increasing the acceptability of and interest in breast cancer risk assessment. | *Thank you this comment, we have added the following:*  *In addition, the findings may be subject to participation bias, as women who chose to take part may have had a greater pre-existing interest in breast cancer and breast health than those who did not participate. This could have contributed to higher levels of acceptability of, and interest in, breast cancer risk assessment than may be observed in the wider population.* | *Pg. 37, lines 790-794.* |
|  | Methods/Discussion - As noted by Braun and Clarke in 2019 ([https://www.tandfonline.com/doi/full/10.1080/2159676X.2019.1628806 [tandfonline.com]](https://urldefense.com/v3/__https:/www.tandfonline.com/doi/full/10.1080/2159676X.2019.1628806__;!!PDiH4ENfjr2_Jw!CeCeLkRRY7N47WBssE3VNQoKolSVPsroGIR8LwHJ5WKo0YdvCMyIpbK2PbUTAH2PPyveSqr0yr_6hIn1RZA9UtOWRA$)), “Assumptions and positionings are always part of qualitative research.”, and they “highlight the importance of methodological scholars locating their stance and acknowledging their position(s)”. In this vein, it may be helpful for the authors to include a statement on their position related to breast cancer risk assessment, especially for the focus group facilitator(s) and team members involved in the analysis, and whether/how this might have influenced participants’ contributions (e.g. perceiving researcher positions and leading to some social desirability bias in responses), and the generated codes and themes. Especially if specific steps were taken to minimise such influences, it would be helpful for the field to learn from this example. | *Thank you for this comment. We have add the following reflexivity statement to the discussion:*  ***Research team reflexivity statement***  The multidisciplinary research team had expertise spanning clinical practice, healthcare, qualitative methods, applied health research, and breast cancer risk research. The focus groups and interview were conducted by VGW, HM, LMcW, MV and FSD. VGW, LMcW HM, and MV are White women, FSD is a Latin American woman. These researchers all have extensive experience in qualitative research methods. VGW and LMcW have particular expertise in breast cancer risk research and engaging under-served and minoritised communities in health research. HM has experience working in applied healthcare research with individuals with mental and physical health conditions in community and clinical settings. MV, LMcW and FSD have experience working with individuals with chronic conditions, including a range of mental health conditions.  As researchers, we could largely be considered outsiders to the communities involved in this study. To address this, the team worked closely with community gatekeepers, charities, and local organisations throughout the research process to build trust, strengthen community understanding, and support meaningful engagement. However, for the mental health group, there was no opportunity to engage with relevant charities during the planning stage. As the researcher who conducted the focus group and interview (MV) had previous professional experience working with young people and individuals living with chronic conditions, this experience informed their approach to facilitating discussions with participants. In particular, the researcher was mindful of avoiding judgemental responses to discussions of lifestyle habits (e.g., alcohol consumption), using sensitive and non-stigmatising language, and avoiding potentially loaded terms such as *illness* where appropriate.  The research teams’ positionality may have also influenced how women’s experiences and perspectives were interpreted during data collection and analysis, with the potential for some cultural or contextual nuances to be overlooked or misinterpreted. To enhance the credibility of the findings, the lead researchers’ interpretations were discussed and reviewed across the multidisciplinary research team throughout the analytic process, enabling reflexive consideration of alternative explanations and perspectives. In addition, preliminary findings were shared with women involved in the subsequent phase of the research, who reported that the findings resonated with their experiences and views. | *Pg. 37-38, lines 801-830.* |
| **Reviewer 2** | I am honored to have had the opportunity to review and evaluate this research work.  Based on the quality of the presented work and the effort invested, I am pleased to express my appreciation and acceptance of this research. I hope the authors continue their academic journey with success and achieve further accomplishments in the future. | *Thank you for such positive feedback. We are so pleased that you appraised our research as good quality and the manuscript an honour to read.* | *NA* |
