# Supplementary material for: Acceptability of breast cancer risk assessment for women aged 30–49: Views of women from six under-served groups
Source: PLoS One. 2026 Aug 17;21(8):e0356263. doi: 10.1371/journal.pone.0356263 (PMC13480479; doi:10.1371/journal.pone.0356263)
Supplement: Peer review history

# Peer review: Decision Letter 0

**DOI:** 10.1371/journal.pone.0356263.r001

**Authors:** Maria Berghs

17 Jun 2026

Dear Dr. French,

Thank you for submitting your manuscript to PLOS ONE. After careful consideration, we feel that it has merit but does not fully meet PLOS ONE’s publication criteria as it currently stands. Therefore, we invite you to submit a revised version of the manuscript that addresses the points raised during the review process.

Please submit your revised manuscript by Aug 01 2026 11:59PM. If you will need more time than this to complete your revisions, please reply to this message or contact the journal office at plosone@plos.org. When you're ready to submit your revision, log on to https://www.editorialmanager.com/pone/ and select the 'Submissions Needing Revision' folder to locate your manuscript file.

- A letter that responds to each point raised by the academic editor and reviewer(s). You should upload this letter as a separate file labeled 'Response to Reviewers'.
- A marked-up copy of your manuscript that highlights changes made to the original version. You should upload this as a separate file labeled 'Revised Manuscript with Track Changes'.
- An unmarked version of your revised paper without tracked changes. You should upload this as a separate file labeled 'Manuscript'.

If you would like to make changes to your financial disclosure, please include your updated statement in your cover letter. Guidelines for resubmitting your figure files are available below the reviewer comments at the end of this letter.

If applicable, we recommend that you deposit your laboratory protocols in protocols.io to enhance the reproducibility of your results. Protocols.io assigns your protocol its own identifier (DOI) so that it can be cited independently in the future. For instructions see: https://journals.plos.org/plosone/s/submission-guidelines#loc-laboratory-protocols. Additionally, PLOS ONE offers an option for publishing peer-reviewed Lab Protocol articles, which describe protocols hosted on protocols.io. Read more information on sharing protocols at https://plos.org/protocols?utm_medium=editorial-email&utm_source=authorletters&utm_campaign=protocols.

As the corresponding author, your ORCID iD is verified in the submission system and will appear in the published article. PLOS supports the use of ORCID, and we encourage all coauthors to register for an ORCID iD and use it as well. Please encourage your coauthors to verify their ORCID iD within the submission system before final acceptance, as unverified ORCID iDs will not appear in the published article. *Only*  the individual author can complete the verification step; PLOS staff *cannot*  verify ORCID iDs on behalf of authors.

We look forward to receiving your revised manuscript.

Kind regards,

Maria J Berghs, PhD

Academic Editor

PLOS One

Journal Requirements:

When submitting your revision, we need you to address these additional requirements.

1.Please ensure that your manuscript meets PLOS ONE's style requirements, including those for file naming. The PLOS ONE style templates can be found at

https://journals.plos.org/plosone/s/file?id=wjVg/PLOSOne_formatting_sample_main_body.pdf and

https://journals.plos.org/plosone/s/file?id=ba62/PLOSOne_formatting_sample_title_authors_affiliations.pdf

2. Thank you for stating the following financial disclosure:

“This study was funded by the Cancer Research UK International Early Detection of Cancer Alliance (ACED) (EDDAMC-2023/100004) and the Manchester Academic Health Science Centre (MAHSC): Cancer Domain. DPF, LMW and HM are supported by the National Institute for Health Research Manchester Biomedical Research Centre (NIHR203308). DF is a NIHR Senior Investigator (NIHR305827). LMW is supported by a Cancer Research UK Career Development Fellowship (RCCCDF-May25/100006). The views expressed are those of the author(s) and not necessarily those of the NIHR or the Department of Health and Social Care.”

Please state what role the funders took in the study.  If the funders had no role, please state: "The funders had no role in study design, data collection and analysis, decision to publish, or preparation of the manuscript."

If this statement is not correct you must amend it as needed.

Please include this amended Role of Funder statement in your cover letter; we will change the online submission form on your behalf.

3. In the online submission form, you indicated that your data is available only on request from a third party. Please note that your Data Availability Statement is currently missing the name of the third party contact or institution / contact details for the third party, such as an email address or a link to where data requests can be made. Please update your statement with the missing information.

4. Please include captions for your Supporting Information files at the end of your manuscript, and update any in-text citations to match accordingly. Please see our Supporting Information guidelines for more information: http://journals.plos.org/plosone/s/supporting-information.

5. If the reviewer comments include a recommendation to cite specific previously published works, please review and evaluate these publications to determine whether they are relevant and should be cited. There is no requirement to cite these works unless the editor has indicated otherwise.

6. Please review your reference list to ensure that it is complete and correct. If you have cited papers that have been retracted, please include the rationale for doing so in the manuscript text, or remove these references and replace them with relevant current references. Any changes to the reference list should be mentioned in the rebuttal letter that accompanies your revised manuscript. If you need to cite a retracted article, indicate the article’s retracted status in the References list and also include a citation and full reference for the retraction notice.

Additional Editor Comments:

The reviewers were very effusive in their praise for the study. Reviewer 1 gives some useful feedback to clarify sample and methodology. I would also follow up with more clarity with respect to reflection of the strengths and weaknesses of the study. This would add rigor and strengthen further.

[Note: HTML markup is below. Please do not edit.]

Reviewer's Responses to Questions

**Comments to the Author**

1. Is the manuscript technically sound, and do the data support the conclusions?

Reviewer #1: Yes

Reviewer #2: Yes

**********

2. Has the statistical analysis been performed appropriately and rigorously?

Reviewer #1: N/A

Reviewer #2: Yes

**********

3. Have the authors made all data underlying the findings in their manuscript fully available?

The PLOS Data policy

Reviewer #1: No

Reviewer #2: Yes

**********

4. Is the manuscript presented in an intelligible fashion and written in standard English?

Reviewer #1: Yes

Reviewer #2: Yes

**********

Reviewer #1: Woof and colleagues’ study provides important insights on the acceptability of breast cancer risk assessment for women aged 30–49 among women from different under-served groups. It is superbly written, with very clear methods, results, and a great discussion. The study is of very high quality and was a pleasure to review.

So I only have very minor comments, listed below.

Results

- lines 244-248: While acknowledging intersectionality, it would be helpful to indicate the number of participants in each of the focus groups.

- Table 1: for clarity, it would be helpful to provide the total numbers of participants who matched to each of the five communities/background listed in the Methods (lines 108-115) in this table, noting some participants may be part of multiple groups. These numbers depend on the definition of some of these groups, so are currently not fully clear from the table.

Discussion

- Strengths and limitations: it may be worth noting that the results could be subject to some participation bias, in that those who chose to participate in the study could have stronger general interest in breast cancer, increasing the acceptability of and interest in breast cancer risk assessment.

Methods/Discussion

- As noted by Braun and Clarke in 2019 (https://www.tandfonline.com/doi/full/10.1080/2159676X.2019.1628806), “Assumptions and positionings are always part of qualitative research.”, and they “highlight the importance of methodological scholars locating their stance and acknowledging their position(s)”. In this vein, it may be helpful for the authors to include a statement on their position related to breast cancer risk assessment, especially for the focus group facilitator(s) and team members involved in the analysis, and whether/how this might have influenced participants’ contributions (e.g. perceiving researcher positions and leading to some social desirability bias in responses), and the generated codes and themes. Especially if specific steps were taken to minimise such influences, it would be helpful for the field to learn from this example.

Reviewer #2: Dear Corresponding author

I am honored to have had the opportunity to review and evaluate this research work.

Based on the quality of the presented work and the effort invested, I am pleased to express my appreciation and acceptance of this research. I hope the authors continue their academic journey with success and achieve further accomplishments in the future.

**********

what does this mean?). If published, this will include your full peer review and any attached files.

If you choose “no”, your identity will remain anonymous but your review may still be made public.

**Do you want your identity to be public for this peer review?**  For information about this choice, including consent withdrawal, please see our Privacy Policy

Reviewer #1: No

Reviewer #2: No

**********

[NOTE: If reviewer comments were submitted as an attachment file, they will be attached to this email and accessible via the submission site. Please log into your account, locate the manuscript record, and check for the action link "View Attachments". If this link does not appear, there are no attachment files.]

To ensure your figures meet our technical requirements, please review our figure guidelines: https://journals.plos.org/plosone/s/figures

You may also use PLOS’s free figure tool, NAAS, to help you prepare publication quality figures: https://journals.plos.org/plosone/s/figures#loc-tools-for-figure-preparation.

NAAS will assess whether your figures meet our technical requirements by comparing each figure against our figure specifications.

# Author comment: Author response to Decision Letter 1

**DOI:** 10.1371/journal.pone.0356263.r002

13 Jul 2026

We have attached a detailed response to reviewer and editor comments in a separate Word file

Attachment Submitted filename: PONE-D-26-00789_R1_Response to Reviewers 13july2026.docx

# Peer review: Decision Letter 1

**DOI:** 10.1371/journal.pone.0356263.r003

**Authors:** Taiwo Opeyemi Aremu

2 Aug 2026

Acceptability of breast cancer risk assessment for women aged 30–49: views of women from six under-served groups

PONE-D-26-00789R1

Dear Dr. French,

We’re pleased to inform you that your manuscript has been judged scientifically suitable for publication and will be formally accepted for publication once it meets all outstanding technical requirements.

Within one week, you’ll receive an e-mail detailing the required amendments. When these have been addressed, you’ll receive a formal acceptance letter and your manuscript will be scheduled for publication.

An invoice will be generated when your article is formally accepted. Please note, if your institution has a publishing partnership with PLOS and your article meets the relevant criteria, all or part of your publication costs will be covered. Please make sure your user information is up-to-date by logging into Editorial Manager at Editorial Manager® and clicking the ‘Update My Information' link at the top of the page. For questions related to billing, please contact billing support.

If your institution or institutions have a press office, please notify them about your upcoming paper to help maximize its impact. If they’ll be preparing press materials, please inform our press team as soon as possible -- no later than 48 hours after receiving the formal acceptance. Your manuscript will remain under strict press embargo until 2 pm Eastern Time on the date of publication. For more information, please contact onepress@plos.org.

Kind regards,

Taiwo Opeyemi Aremu, MD, MPH, PhD

Academic Editor

PLOS One

Additional Editor Comments (optional):

Reviewers' comments:

Reviewer's Responses to Questions

**Comments to the Author**

Reviewer #2: (No Response)

**********

2. Is the manuscript technically sound, and do the data support the conclusions?

Reviewer #2: Yes

**********

3. Has the statistical analysis been performed appropriately and rigorously?

Reviewer #2: Yes

**********

4. Have the authors made all data underlying the findings in their manuscript fully available?

The PLOS Data policy

Reviewer #2: Yes

**********

5. Is the manuscript presented in an intelligible fashion and written in standard English?

Reviewer #2: Yes

**********

Reviewer #2: I have carefully reviewed this manuscript and found it to be scientifically sound, well organized, and relevant to the journal’s scope. The study addresses an important research question, the methodology is appropriate, and the results are clearly presented and supported by the data. The discussion provides meaningful interpretation and highlights the clinical and scientific significance of the findings. The authors have responded adequately to previous concerns, and the manuscript has improved substantially. Only minor editorial revisions, if any, may be considered during the production stage. Overall, I believe this manuscript meets the standards for publication and recommend its acceptance in its current form.

**********

what does this mean?). If published, this will include your full peer review and any attached files.

If you choose “no”, your identity will remain anonymous but your review may still be made public.

**Do you want your identity to be public for this peer review?**  For information about this choice, including consent withdrawal, please see our Privacy Policy

Reviewer #2: **Yes:**  Engy Ashaat

**********

# Editor report: Acceptance letter

**DOI:** 10.1371/journal.pone.0356263.r004

**Authors:** Taiwo Opeyemi Aremu

PONE-D-26-00789R1

PLOS One

Dear Dr. French,

I'm pleased to inform you that your manuscript has been deemed suitable for publication in PLOS One. Congratulations! Your manuscript is now being handed over to our production team.

At this stage, our production department will prepare your paper for publication. This includes ensuring the following:

* All references, tables, and figures are properly cited

* All relevant supporting information is included in the manuscript submission,

* There are no issues that prevent the paper from being properly typeset

You will receive further instructions from the production team, including instructions on how to review your proof when it is ready. Please keep in mind that we are working through a large volume of accepted articles, so please give us a few days to review your paper and let you know the next and final steps.

Lastly, if your institution or institutions have a press office, please let them know about your upcoming paper now to help maximize its impact. If they'll be preparing press materials, please inform our press team within the next 48 hours. Your manuscript will remain under strict press embargo until 2 pm Eastern Time on the date of publication. For more information, please contact onepress@plos.org.

You will receive an invoice from PLOS for your publication fee after your manuscript has reached the completed accept phase. If you receive an email requesting payment before acceptance or for any other service, this may be a phishing scheme. Learn how to identify phishing emails and protect your accounts at https://explore.plos.org/phishing.

If we can help with anything else, please email us at customercare@plos.org.

Thank you for submitting your work to PLOS ONE and supporting open access.

Kind regards,

PLOS ONE Editorial Office Staff

on behalf of

Dr. Taiwo Opeyemi Aremu

Academic Editor

PLOS One
